# Extracting chemical reactions from text using Snorkel

**DOI:** 10.1186/s12859-020-03542-1

**Published:** 2020-05-27

**Authors:** Emily K. Mallory, Matthieu de Rochemonteix, Alex Ratner, Ambika Acharya, Chris Re, Roselie A. Bright, Russ B. Altman

**Affiliations:** 1 Biomedical Informatics Training Program, Stanford University, Stanford, CA USA; 2 Department of Statistics, Stanford University, Stanford, CA USA; 3 Department of Computer Science, Stanford University, Stanford, CA USA; 4 Office of Health Informatics, Office of the Chief Scientist, Office of the Commissioner, Food and Drug Administration, Silver Spring, MD USA; 5 Departments of Medicine, Genetics, Bioengineering, and Biomedical Data Science, Stanford University, Stanford, CA USA

**Keywords:** Text mining, Chemical reactions, Snorkel, Curation, Database

## Abstract

**Background:**

Enzymatic and chemical reactions are key for understanding biological processes in cells. Curated databases of chemical reactions exist but these databases struggle to keep up with the exponential growth of the biomedical literature. Conventional text mining pipelines provide tools to automatically extract entities and relationships from the scientific literature, and partially replace expert curation, but such machine learning frameworks often require a large amount of labeled training data and thus lack scalability for both larger document corpora and new relationship types.

**Results:**

We developed an application of Snorkel, a weakly supervised learning framework, for extracting chemical reaction relationships from biomedical literature abstracts. For this work, we defined a chemical reaction relationship as the transformation of chemical A to chemical B. We built and evaluated our system on small annotated sets of chemical reaction relationships from two corpora: curated bacteria-related abstracts from the MetaCyc database (MetaCyc_Corpus) and a more general set of abstracts annotated with MeSH (Medical Subject Headings) term Bacteria (Bacteria_Corpus; a superset of MetaCyc_Corpus). For the MetaCyc_Corpus, we obtained 84% precision and 41% recall (55% F1 score). Extending to the more general Bacteria_Corpus decreased precision to 62% with only a four-point drop in recall to 37% (46% F1 score). Overall, the Bacteria_Corpus contained two orders of magnitude more candidate chemical reaction relationships (nine million candidates vs 68,0000 candidates) and had a larger class imbalance (2.5% positives vs 5% positives) as compared to the MetaCyc_Corpus. In total, we extracted 6871 chemical reaction relationships from nine million candidates in the Bacteria_Corpus.

**Conclusions:**

With this work, we built a database of chemical reaction relationships from almost 900,000 scientific abstracts without a large training set of labeled annotations. Further, we showed the generalizability of our initial application built on MetaCyc documents enriched with chemical reactions to a general set of articles related to bacteria.

## Background

Enzymes and the reactions they catalyze are key for understanding how small molecules are processed in the cell. In particular, chemical reactions that occur in the human gut microbiome can shed light on drug mechanism of action and metabolism when these reactions transform drugs or other bio-active small molecules. Since unintentional drug transformations in the human gut can affect drug response and side effects in patients [1], understanding the space of chemical reactions in bacteria is necessary for predicting and cataloging enzymatic transformations of drugs in the human gut microbiome.

Databases such as MetaCyc [2] and KEGG (Kyoto Encyclopedia of Genes and Genomes) [3] contain high quality pathways with metabolic reactions that are manually annotated by human experts. However, manual human annotation restricts the coverage and growth of the database with respect to the biomedical literature. This inability to scale to larger and larger corpora is a limiting factor in large data-driven studies. On the other hand, the biomedical literature is the single best source of known metabolic reactions across all organisms. While rich in information, the computationally inaccessible nature of literature text presents a challenge for relationship extraction.

Extraction of chemical reactions from text requires the extraction of chemical entities and the relationship between them as described in a sentence or paragraph. In natural language processing, the task of entity extraction is referred to as named entity recognition and the task of connecting two entities in a defined relationship is relationship extraction. For biological datasets, text mining tools exist to extract protein-protein [4] and other biomedical associations [5]. While PubTator from the National Center for Biotechnology Information [6] (using the method tmChem [7]) extracts and releases datasets of chemical entities extracted from abstract sentences, to our knowledge no method exists for extracting the primary transformations occurring in chemical reactions from text. In order to build a database of chemical reactions from text, a text mining application framework is needed for rapid development with high accuracy, usability for nontechnical users, and corpus size scalability in order to enable downstream analyses using the resulting extracted relationship database.

Recent advances in natural language processing and word embeddings have introduced new approaches for relationship extraction. Self-supervised word embedding approaches such as word2vec [8] and BERT (Bidirectional Encoder Representations from Transformers) [9] construct vector representations of words based on the context around a word in a given corpus. BERT is a language representational model that has been applied to a number of NLP (natural language processing) tasks, such as named entity recognition and relationship extraction. Building on the original BERT model, Lee et al. released BioBERT - a BERT model trained on biomedical literature from PubMed and PubMed Central [10]. BioBERT achieved between 75 and 77% precision for the task of detecting protein-chemical relationships, a task included in BioCreative VI [11, 12]. While Bio-BERT demonstrated improved performance for relationship extraction of protein-chemical relationships, amongst other relationships, lack of accessible text mining tools and frameworks for domain experts remains a challenge. Additionally, although community challenges have enabled the BioNLP (biomedical natural language processing) community to solve some pressing extraction tasks, a lack of labeled datasets has limited the applicability of these tools to other tasks in within subdomains of biology and medicine.

Snorkel is a framework for creating large training datasets and streamlining the typical natural language processing extraction pipeline for entities and relationships from text [13, 14]. The main advantage for Snorkel is that it does not require hand-labeled datasets that may need to be relabeled, extended, or disregarded when a task or schema changes. Instead, the user only has to focus on designing relevant labeling functions to automatically assign noisy labels. These labeling functions can then be reused for updating or repurposing the literature corpus. As a result, the Snorkel framework allows very fast prototyping and good scalability to large datasets.

Users interact with Snorkel using pre-defined functions for parsing text, detecting candidate entities or relationships (e.g., pair of two entities co-occurring in a sentence), assigning training labels, extracting text-based features, and training a machine learning model for the final prediction. Importantly, users focus primarily on developing **labeling functions** and not constructing large hand-labeled training sets as found in typical machine learning pipelines. These labeling functions encode basic rules for automatically labeling noisy training examples for a combination of generative and discriminative machine learning models in Snorkel. The final output of Snorkel is a binary prediction of a true relationship for each candidate relationship.

The focus of databases such as MetaCyc [2] is high-quality expert curation (in MetaCyc’s case, enzymatic reactions and pathways) at the expense of speed and coverage of biomedical literature. Preliminary results of applying Snorkel to multiple biomedical entity and relationship tasks demonstrated Snorkel’s fast prototyping and scalability for different biomedical tasks. In this work, we built a Snorkel application to extract chemical reaction relationships from the biomedical literature in a higher-throughput and more scalable approach.

## Methods

**Fig. 1:**
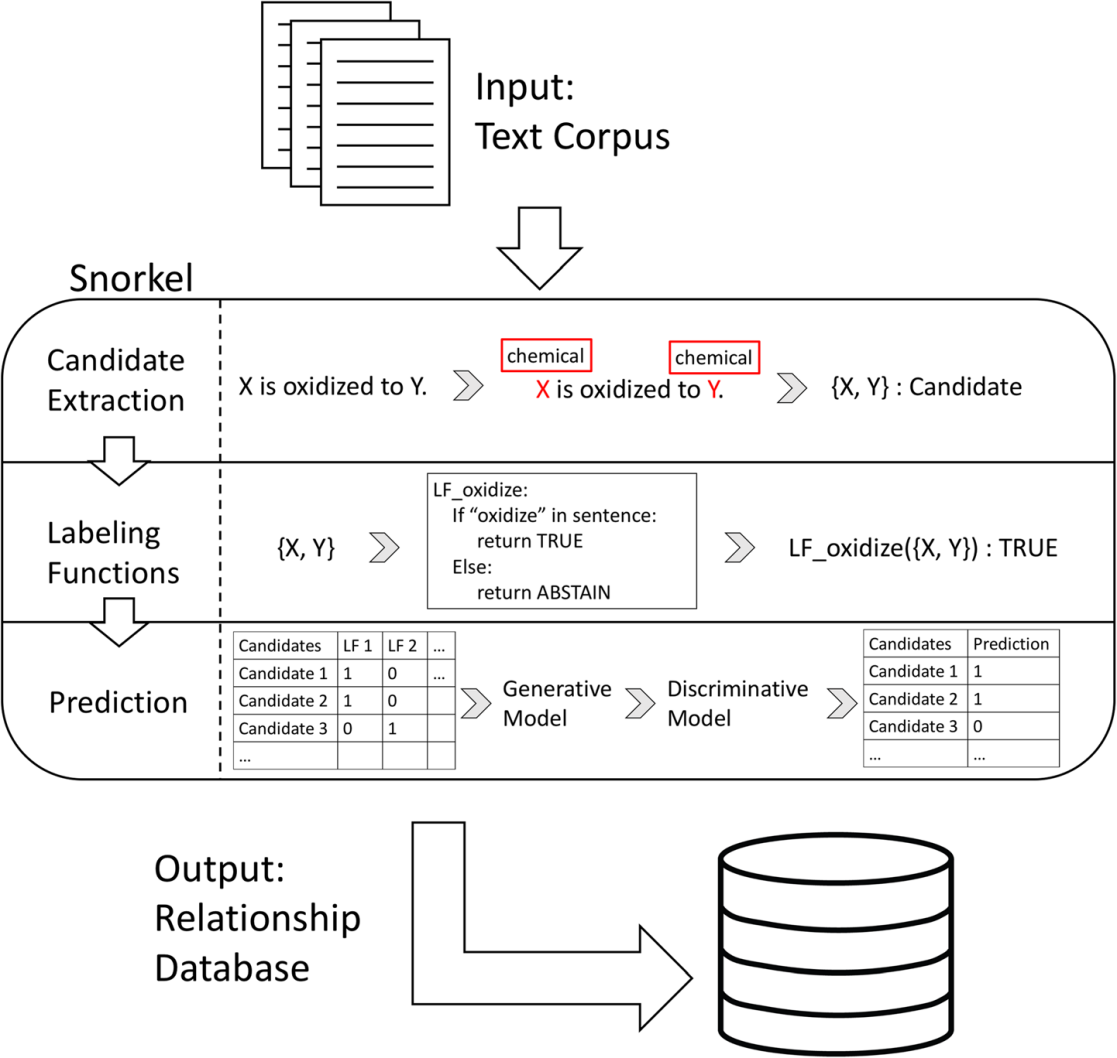
Overview of the Snorkel pipeline. First, users input a text corpus. Snorkel extracts relationships of interest by 1) detecting co-occurring entities (i.e., relationship candidates), 2) applying labeling functions to automatically label noisy training examples, and 3) training generative and discriminative machine learning models using the labeling functions and general features to predict which candidates are true relationships. The output is a binary prediction of a true relationship for each relationship candidate

In this work, we built a knowledge base construction application, using the Snorkel framework, for extracting chemical reaction relationships from biomedical literature abstracts. The Snorkel pipeline is depicted in Fig. 1 and described below. In summary, our application takes a corpus of texts as an input and outputs all co-occurring entity pairs with a binary prediction of a true relationship. Using the Snorkel framework, we primarily focused on two tasks: extracting candidate entities and relationships and designing labeling functions. These labeling functions allow us to apply noisy labels to candidate relationships without labeling large training sets by hand. We next used these labeling functions to train a combination of generative and discriminative machine learning models that generalize beyond the initial noisy labels.

### Datasets

#### MetaCyc_Corpus

We built and evaluated the Snorkel application using a corpus of 1799 PubMed abstracts curated for enzymatic chemical reactions in bacteria by MetaCyc [2] in version 20.0 (downloaded August 2016). We parsed the corpus using the Snorkel pipeline and the SpaCy parser [15] to detect sentences, word tokens, parts-of-speech tags and dependency graphs. We included the titles of the abstracts in the database. In addition, we included PubTator Chemical annotations [6] for all abstracts in the corpus. We refer to this corpus as the MetaCyc_Corpus.

#### Bacteria_Corpus

To generalize and expand the MetaCyc_Corpus, we built a second dataset using the following PubMed query:

bacteria [MeSH Terms] AND has abstract [filter] AND English [language]

This query included all abstracts in English, annotated with the MeSH term “bacteria”, as of Feb 2018. We filtered these abstracts to the subset containing PubTator Chemical annotations (retrieved March 2018). From the 880,134 abstracts returned from the Pubmed query, we included 873,237 abstracts with PubTator Chemical annotations in the abstract corpus. We refer to this corpus as the Bacteria_Corpus.

To facilitate comparison of the results between the two datasets, we added all the documents from the MetaCyc_Corpus that were available in the PubTator dump to the Bacteria_Corpus.

### Candidate extraction

The primary task of relationship extraction in Snorkel is to extract pairs of entities that fit some interaction or relationship definition. This relationship definition defines a **candidate relationship**, a pair of entities that may participate in a true relationship. Importantly, this definition should have high recall for true relationships but may have low precision. For the task of extracting chemical reaction relationships, we want to define a relationship definition to capture the majority of chemical reactions present in literature abstracts with a focus on recall.

The following is an example sentence describing a chemical reaction:

X is converted into Y in the absence of Z.

There is one true chemical reaction in this sentence: X is converted into Y. In this example, the task of the Snorkel pipeline is to extract the pair of words (X, Y).

We define a chemical reaction relationship **candidate** as an ordered pair of distinct chemicals co-occurring in a sentence. In our example, there are three chemicals in the sentence: X, Y, and Z. Therefore, there are six candidate chemical reactions: (X, Y), (X, Z), (Y, X), (Y, Z), (Z, X), and (Z, Y).

We performed candidate extraction on both datasets using Chemical entity tags from PubTator and a candidate extraction workflow with objects and functions built into Snorkel. In summary, we scanned sentences in both corpora for co-occurring PubTator chemical entities to create candidate relationships. As described above, we created two candidate relationships for every pair of entities to capture the correct order of the potential chemical reaction. This extraction procedure yielded a large number of candidates (for a sentence with n chemicals, we have n(n-1) candidates). Candidate counts are reported in Table 1.

**Table 1:** Number of abstracts and candidates in MetaCyc_Corpus and Bacteria_Corpus

Corpus	Abstracts	Candidates
MetaCyc_Corpus	1799	67,922
Bacteria_Corpus	873,237	8,936,941

It is important to note that the Bacteria_Corpus is 485 times larger than the MetaCyc_Corpus in terms of the number of documents, but it is only 132 times larger in terms of candidates. The documents in the MetaCyc_Corpus have on average 3.69 more candidates and are understandably enriched for sentences that discuss chemical reactions.

### Learning procedure

Candidate extraction produces a set of candidate chemical reaction relationships. During learning, we trained machine learning models to predict which candidates were true chemical reactions (Fig. 1, steps 2 and 3). We first designed labeling functions to automatically label noisy training examples. Next, we trained a generative model to learn the inaccuracies of the labeling functions. Finally, we trained a discriminative model using the scores from the generative model as training labels to predict true chemical reactions.

#### Labeling functions

Labeling functions have three important features:

- Labeling functions are context-aware: they may rely on sentence or document-level patterns, use the entity tags or labels, or even use external information.
- Labeling functions can be very general and correspond to a *weak* signal, or they can be very specific and handle special, uncommon cases.
- Labeling functions have 3 possible outputs: TRUE, FALSE, or ABSTAIN.

We designed **unipolar** labeling functions, that only have two possible outputs. The positive labeling functions select either TRUE or ABSTAIN, while the negative labeling functions select either FALSE or ABSTAIN. These unipolar labeling functions deviate from prior work [14] in order to handle large class imbalances. Importantly, labeling functions must only provide noisy information about the likelihood of a chemical reaction and need not be individually very predictive. For the MetaCyc_Corpus, we designed five positive labeling functions and 11 negative functions. Example positive and negative labeling functions are detailed in Table 2. Descriptions of all labeling functions are listed in Supplemental Data Section 1.7.

**Table 2:** Example labeling functions for the MetaCyc corpus

Example labeling function	Description
LF_keyword_context	If there is a word of a given list, such as *reduce*, *oxidize*, *transform*, or *afford* between the two words, we label TRUE
LF_sep_verb	If the chemicals are separated by a verb, we label TRUE
LF_argument_order	If the candidate product is before the candidate substrate, we label FALSE
LF_followed_ase	If one of the chemicals is followed by a word that ends with “ase”, we label FALSE
LF_sep_or	If the chemicals are separated by the word *or*, we label FALSE

Because the Bacteria_Corpus is over 100 times larger than the MetaCyc_Corpus and had a larger variety of candidates, we designed an extra set of labeling functions to capture the complexity and diversity of this dataset. Table 3 contains examples of additional labeling functions for the Bacteria_Corpus. We added five additional negative labeling functions and one positive, to obtain an overall set of 22 labeling functions for the Bacteria_Corpus. For LF_metacyc, we used <substrate, product> pairs from enzymatic reactions from MetaCyc (version 20.0). We limited these reactions to include only 1:1 substrate-product transformations, after filtering proton acceptors and donors, water molecules, and hydrogen atoms.

**Table 3:** Examples of additional labeling functions on the Bacteria_Corpus

Example labeling function	Description
LF_metacyc	If the chemical reaction is already in the MetaCyc curated database, we label TRUE
LF_chemical_elements	If one of the chemicals is a chemical element, we label FALSE
LF_group	If there is a close mention of a functional chemical group, we label FALSE
LF_treatment	If there is mention of keywords frequently associated with clinical trials, we label FALSE

#### Generative model

After applying the labeling functions to the candidate relationships, we trained a generative model to learn the inaccuracies of the labeling functions and assign a probabilistic score to each candidate. From the labeling function step, the labels (from the labeling functions) for the extracted candidates form a sparse matrix of 0,1 and − 1 (ABSTAIN, TRUE, and FALSE, respectively). The purpose of the generative model is to infer an underlying distribution from which those labels have been sampled, and to use this model to score all the candidates from 0 to 1 (0 being a negative relationship and 1 a positive relationship). We refer to these scores as the **training marginals.** The training marginals formed the training example labels to the discriminative model (described below).

We used different versions of the generative model implementation for the MetaCyc_Corpus and Bacteria_Corpus. The current released version of Snorkel uses a Gibbs Sampling method, that has been thoroughly tested and evaluated by the Snorkel developers [13, 14]. We used this version for the MetaCyc_Corpus. However, this implementation is not robust to unbalanced sets of unipolar labeling functions and heavy class imbalance in the dataset.

Because the Bacteria_Corpus has a very high class imbalance and unbalanced set of unipolar labeling functions, we used a new class-conditional matrix completion based approach of the generative model that is much more robust to the imbalance [16]. As this implementation is not needed on the MetaCyc_Corpus, we preferred keeping the Gibbs Sampling implementation when possible (see Supplemental Section 1.6 for comparison of the two generative model approaches).

#### Discriminative model

Whereas the generative model provides probabilistic labels for each candidate using the labeling functions, the discriminative model generalizes beyond the labeling functions by using the training marginals as training example labels.

We trained a discriminative model using the training marginals from the generative model as training labels. The discriminative model is a logistic regression model that uses a set of *default* NLP features (n-grams, etc...) computed on the training set. We trained the discriminative model using the sigmoid cross-entropy loss for binary classification, with an elastic net penalty. Before training the discriminative model, we resampled the dataset (see Supplemental Section 1.1 for the detailed procedure and Supplemental Section 1.5 for comments). The hyperparameters for the discriminative model and the resampling were chosen using a grid search on a development set with a small number of labeled candidate chemical reaction relationships (described in Section Evaluation Framework).

### Evaluation framework

One of the main advantages of the Snorkel pipeline is that we do not need to manually annotate training data and instead rely on the labeling functions and a small annotated development set and held-out test set. For performance evaluation, as well as labeling function and model design, we curated chemical reaction relationship candidates in a subset of abstracts from the two chemical reaction corpora.

#### Evaluation datasets for MetaCyc_Corpus

To achieve proper reproducibility and evaluation of the performance, we split the MetaCyc_Corpus into three distinct splits of the data.

- MetaCyc_Train: constituted the majority of the abstracts. We trained the models on this split only. However, unlike the training sets in usual machine learning pipelines, the candidates have not been manually annotated.
- MetaCyc_Dev: a small development subset of the initial corpus used to tune the model parameters using gridsearch and design the labeling functions
- MetaCyc_Test: a subset of the corpus held out from model training and development to evaluate the out-of-sample model performance.

We randomly sampled abstracts without replacement from the full corpus to create MetaCyc_Dev and MetaCyc_Test and curated the associated candidates. Table 4 reports the number of abstracts, candidates, and positive examples for the train, development and test sets.

**Table 4:** Sizes and gold label statistics of the three splits for the MetaCyc_Corpus

Split	Abstracts	Candidates	Positives	Docs w. candidates	Docs w. positives
MetaCyc_Train	1753	65,398	–	1544	–
MetaCyc_Dev	23	1292	60	23	16
MetaCyc_Test	23	1232	51	23	15

#### Evaluation datasets for Bacteria_Corpus

We built the Bacteria_Corpus by extending the MetaCyc_Corpus. The extension of the dataset created a need to have a new test set to evaluate the performance of the models on the updated task. However, we kept MetaCyc_Test as a test set to have an estimator of the model performance on the MetaCyc_Corpus (that is a subset of the Bacteria_Corpus). It is also necessary to keep MetaCyc_Dev, as it has been used to design a majority of the labeling functions.

As a result, the Bacteria_Corpus experiment relied on a **four-split architecture**, divided as follows:

- Bacteria_Train: the training set, constituted the majority of the abstracts
- Bacteria_Test: a subset of the corpus held out from model training and development to evaluate the out-of-sample model performance
- Bacteria_Dev: MetaCyc_Dev, augmented with 200 abstracts randomly sampled from the new documents in the Bacteria_Corpus to gridseach the models and design the labeling functions.
- MetaCyc_Test: included with no change.

We randomly sampled abstracts without replacement from the full corpus to create Bacteria_Test and Bacteria_Dev and curated all candidates in the sampled abstracts. Table 5 reports the number of abstracts, candidates, and positive examples for the different splits of the Bacteria_Corpus. The 400 documents added to Bacteria_Test and Bacteria_Dev were randomly sampled from the documents that were not already in MetaCyc_Dev or MetaCyc_Test. Therefore, we developed the final models on Bacteria_Dev and evaluated performance on both Bacteria_Test and MetaCyc_Test.

**Table 5:** Sizes and gold label statistics of the splits for the Bacteria_Corpus

Split	Abstracts	Candidates	Positives	Docs w. candidates	Docs w. positives
Bacteria_Train	872,591	8,928,937	–	417,404	–
Bacteria_Test	200	2398	43	96	13
Bacteria_Dev	223	2806	69	110	22
MetaCyc_Test	23	1212	49	23	15

Due to minor updates in the candidate extraction process, and in the PubTator chemical tags, there is < 1% discrepancy in the number of candidates extracted between the MetaCyc and the Bacteria experiments for the 1791 abstracts that are in both corpus.

#### Evaluation metrics

We evaluated the performance of three prediction models: majority voting of the labeling functions, generative model only, and discriminative model using the training marginals from the generative model (henceforth referred to as discriminative model for simplicity). We computed precision, recall, and F_1_ score using the development and held-out test sets for MetaCyc_Corpus and Bacteria_Corpus.

$$ Precision:P=\frac{TP}{TP+ FP} $$

$$ Recall:R=\frac{TP}{TP+ FN} $$

$$ {F}_1 score:\frac{2P\ast R}{P+R} $$

Where:

- TP (True Positives): Positive examples, correctly classified
- TN (True Negatives): Negative examples, correctly classified
- FP (False Positives): Negative examples, misclassified
- FN (False Negatives): Positive examples, misclassified

We also used the F_β_ score to select the models in the gridsearch, as it allows to shift the precision-recall trade-off towards more precision or more recall if needed.

The F_β_ score is defined as $\left(1+{\beta}^2\right)\frac{P\ast R}{\beta^2P+R}$.

## Results

We built and evaluated the Snorkel application for extracting chemical reaction relationships from two datasets: MetaCyc_Corpus and Bacteria_Corpus.

### Comparison of labeling function coverage

An important component of the labeling function design process is to analyze the statistics of the different labeling functions. Table 6 provides example labeling functions with proportion coverage, overlaps, and conflicts in the MetaCyc_Corpus and the Bacteria_Corpus. For MetaCyc_Corpus, there are both very wide labeling functions and very precise ones. For example, LF_argument_order has a 0.50 coverage or proportion of candidates labeled by the labeling function because of its generality. However, LF_keyword_context only labels rare candidates and therefore has a low coverage. The coverage of the labeling functions initially designed for the MetaCyc_Corpus dropped when we applied the labeling functions to the Bacteria_Corpus. For example, the coverage of LF_followed_ase dropped from 0.18 to 0.015.

**Table 6:** Labeling Function metrics for MetaCyc_Corpus and Bacteria_Corpus. Coverage refers to the proportion of candidates labeled with the labeling function. Overlaps refers to the proportion of candidates labeled with another labeling function. Conflicts refers to proportion of candidates labeled with an opposing labeling function

	MetaCyc_Corpus			Bacteria_Corpus		
Labeling function	Coverage	Overlaps	Conflicts	Coverage	Overlaps	Conflicts
LF_keyword_context	0.005963	0.002110	0.001361	0.001902	0.001750	0.001719
LF_sep_verb	0.000933	0.000291	0.000092	0.001252	0.001146	0.001137
LF_argument_order	0.500000	0.238234	0.005520	0.499939	0.470721	0.016873
LF_followed_ase	0.180954	0.155800	0.001697	0.015969	0.015838	0.000966
LF_sep_or	0.006453	0.003333	0.000000	0.006702	0.006399	0.000526
LF_metacyc	–	–	–	0.031805	0.030915	0.030823
LF_chemical	–	–	–	0.130835	0.130539	0.004871
LF_treatment	–	–	–	0.029490	0.028674	0.000609

### Evaluation on the MetaCyc_Corpus

We evaluated Snorkel for the task of extracting chemical reaction relationships from text. Table 7 contains the evaluation result using majority voting, generative model, and discriminative model on the MetaCyc_Corpus. The generative model performed similarly to majority voting. However, the discriminative model brought a significant lift of the performance and increased precision from 0.79 to 0.84 for the discriminative model. In addition, there was a 19 point increase in recall from 0.22 to 0.41.

**Table 7:** Evaluation results for MetaCyc_Corpus. We evaluated three models: majority voting, generative model, and discriminative model

Model	Coverage	Precision	Recall	F1 Score
Majority voting	0.73	0.79	0.22	0.34
Generative model	0.73	0.79	0.22	0.34
Discriminative model	**1.00**	**0.84**	**0.41**	**0.55**

### Evaluation on the Bacteria_Corpus

We evaluated Snorkel for the task of extracting chemical reaction relationships from text. Table 8 contains the evaluation result using majority voting, generative model, and discriminative model on the Bacteria_Corpus using MetaCyc_Test. We used a threshold of 0.50 on the final score given by the discriminative model to issue predictions.

**Table 8:** Evaluation results on the Bacteria_Corpus, using a 0.5 threshold. We evaluated three models: majority voting, generative model, and discriminative model

Model	Coverage	Precision	Recall	F1 Score
Majority voting	0.92	**1.00**	0.20	0.34
Generative model	0.94	**1.00**	0.33	0.49
Discriminative model	**1.00**	0.50	**0.61**	**0.55**

The generative model brings a significant lift on the recall for Bacteria_Corpus, increasing recall 12 points from 0.20 to 0.33 while preserving the precision. The discriminative model changes the precision-recall tradeoff towards a more balanced prediction, that lifts the F_1_ score by six points for the discriminative model compared to the generative model. While precision decreased to 0.50, recall increased to 0.61.

In total, we extracted 6871 chemical reaction relationships from almost nine million candidates in Bacteria_Corpus. This total includes both chemical reaction relationships from MetaCyc_Corpus and Bacteria_Corpus.

## Discussion

We designed a Snorkel application to construct a chemical reaction database from text using a small corpus of abstracts enriched with enzymatic chemical reactions and extended this application to a more general corpus of almost a million abstracts broadly related to bacteria. The ability to generate relationship data from domain-specific literature is essential to creating rich datasets for improving large data-driven tasks. We defined a chemical reaction relationship for extraction; however, we note that our approach is much more easily modified to select different (e.g. more specific) relation types of interest, as this only requires modifying code, rather than relabeling datasets by hand as in traditional approaches. In addition, our results can be used to develop Snorkel extensions to increase accuracy for complex chemical reaction and chemistry literature.

This framework substantially changes how one approaches text mining applications and development, and considerably speeds up the extractor creation process. Importantly, the focus on designing labeling functions in Snorkel allows for quicker development time and scalability to larger and more general datasets. We developed 16 labeling functions for labeling almost 68,000 candidates from MetaCyc_Corpus and added only six new labeling functions to label almost nine million candidates from Bacteria_Corpus. These labeling functions included high precision/low recall functions such as *if two chemicals are separated by a sequence of words that contains oxidized to, reduced to, conversion to, label candidate TRUE*. This labeling function labels candidates that are likely correct (i.e., marginal probability close to 1) but will miss many candidates that do not have straightforward syntax describing the relationship. Other labeling functions will be less specific but label many candidates either True or False. For example, a labeling function that returns True if the second chemical follows the first chemical in the sentence labeled 50% of all candidates in both MetaCyc_Corpus and Bacteria_Corpus. However, a labeling function that returns True if a verb separates two chemicals in a sentence labeled a smaller subset of the data (0.09 and 0.1% for MetaCyc_Corpus and Bacteria_Corpus, respectively). While precision decreased compared to MetaCyc_Corpus, Bacteria_Corpus included a more diverse set of documents (all documents related to bacteria using MeSH terms) which in turn included a more diverse chemical set. Thus while Bacteria_Corpus included more false positive chemical reaction relationships, we increased recall substantially to capture more *true* chemical reaction relationships. The boost in recall from generative to discriminative model allowed for more generalization in the Bacteria_Corpus. The focus on precision vs recall is a design decision that depends partially on downstream application use.

From almost nine million candidates in Bacteria_Corpus, we extracted 6871 chemical reaction relationships. These reactions included simple substrate-product pairs. For example, we extracted the reaction *gluconic acid* to *ethanol* from the sentence “We report on engineering *Escherichia coli* to produce ethanol at high yield from gluconic acid [17].” We also extracted reactions where multiple substrates and/or products were mentioned in the sentence. For example, Snorkel extracted the hydrolysis of *naproxen nitrile* to *S-naproxen* from the sentence “Enantioselective hydrolysis of racemic naproxen nitrile and naproxen amide to S-naproxen by new bacterial isolates [18].” The sentence “These results suggest that HMF can be metabolically activated to an allylic sulfuric acid ester which may play a role as an ultimate electrophilic metabolite in toxification of the parent compound in vivo.” from [19] presents an interesting example. Here, we extracted HMF goes to allylic sulfuric acid ester. While we know the starting compound, we do not know the exact product. However, these are important cases to capture since chemistry literature can be vague on the chemical structure and name depending on when the article was written.

While we extracted chemical reaction relationships from clear substrate to product transformations, Snorkel struggled to correctly detect negative reactions when co-occurring with a positive reaction. For example, the sentence “Only D-cysteine but not L-cysteine was converted by D-CDes to pyruvate, H2S, and NH3.” [20] includes multiple potential substrate and product pairs. However, only one of the potential substrates is a true substrate in the described reaction (D-cysteine). Snorkel extracted all pairs of the potential substrates D-cysteine and L-cysteine with the products pyruvate, H2S, and NH3. The resulting extracted reactions include the correct reaction pairs (D-cysteine, pyruvate), (D-cysteine, H2S), and (D-cysteine, NH3) but also incorrect reaction pairs (L-cysteine, pyruvate), (L-cysteine, H2S), and (L-cysteine, NH3). Thus in this sentence, 50% of extractions were incorrect. It is important to note that none of the substrates were extracted together in an incorrect or false chemical reaction, and neither were the potential products. Additional curated candidates in the development and test sets, along with further development of the labeling functions, would provide additional signal to detect negative candidates co-occurring with positive candidates correctly.

Class imbalance within and between the two corpora was a key challenge of this work. An estimation of the proportion of positive candidates can be done based on the number of positive candidates in MetaCyc_Dev (reported in Table 4). This reveals a high class imbalance (5%). Table 4 also shows that 88% of the documents have at least a candidate (decreases to 47% in Bacteria_Corpus). However, the labeled subsets suggest that only 67% of the documents that have a candidate have a positive candidate. The class imbalance is higher in the Bacteria_Corpus. The labeled subsets suggest that for the Bacteria_Corpus, only 13% of the documents with a candidate have a positive candidate, which is significantly lower than the MetaCyc_Corpus, especially taking into account that the MetaCyc_Corpus is included in the Bacteria_Corpus. The MetaCyc_Corpus is composed of articles curated for enzymatic reactions and is thus enriched for these types of chemical reactions (see Table 1). The overall proportion of positive candidates drops to roughly 1.5% on the Bacteria_Corpus. This makes the **class imbalance** challenge even more difficult compared to MetaCyc_Corpus.

To address the class imbalance problem, we applied a resampling procedure to subsample the training data used in the discriminative model (described in Supplemental Methods). Additional experiments performed showed that the optimal results were obtained by training on approximately 10% of the dataset (see Supplemental Data). We found that the negative examples were very similar to one another and did not bring much diversity in the training set.

One of the hurdles for more domain and subdomain-specific extraction tools remains the challenge-focused aspect of relationship extraction development. There are well-curated training and evaluation datasets for chemical-disease and protein-protein relationships, amongst others. These datasets arose from community challenges to develop state-of-the-art methods for biomedical entity and relationship extraction (e.g., BioCreative VI) [11]. These community-drive challenges are critical for moving the field forward in terms of method development and solving specific extraction tasks. However, there remains a need for accessible text mining application and/or tool development for domain experts. These users have specific tasks relevant to their research and subfield of biology and medicine. Snorkel allows these domain experts to develop applications for their needs. In this work, we developed a Snorkel application for the task of chemical reaction relationship extraction and demonstrated the labeling function development process for future Snorkel applications.

## Conclusion

We developed a Snorkel application for extraction chemical reaction relationships from literature abstracts using an enriched corpus of chemical reactions and extended the application to a larger and diverse set of abstracts related to bacteria. In total, we extracted chemical reaction relationships from nearly 900,000 abstracts from PubMed related to bacteria. In this work, we showed the first biological application of the Snorkel infrastructure and the scalability of the Snorkel pipeline to very large datasets. This work enables development of future Snorkel extraction tasks and downstream prediction analyses based on enzymatic reaction data.

## Supplementary information

**Additional file 1.** “Extracting Chemical Reactions from Text using Snorkel Supplemental Data”. File contains supplemental methods and results for the main manuscript.

**Additional file 2.** Tab-delimited file contains the list of 6871 extracted bacterial chemical reactions. The supplemental data file contains NCBI PubMed identifiers (i.e., pmids), sentence number, extracted chemicals and character positions in the abstract text, and training marginals for the prediction algorithm. All extracted chemical reactions are predicted as chemical reactions from the discriminative model.

## Data Availability

All data generated or analyzed during this work are included in this published article and its supplementary information files. Supplemental methods and evaluation results are available in “Extracting Chemical Reactions from Text using Snorkel Supplemental Data”. The list of 6871 extracted bacterial chemical reaction relationships is available in a separate tab-delimited supplemental file. The supplemental data file contains NCBI PubMed identifiers (i.e., pmids), sentence number, extracted chemicals and character positions in the abstract text, and training marginals for the prediction algorithm. All extracted chemical reaction relationships are predicted as chemical reactions from the discriminative model. Additional data available upon request. We also provide code and labeling functions at http://simtk.org/projects/chem-rxn

## Abbreviations

BERT: Bidirectional Encoder Representations from Transformers
BioNLP: Biomedical natural language processing
KEGG: Kyoto Encyclopedia of Genes and Genomes
MeSH: Medical Subject Headings
NLP: Natural language processing
