# Supplementary material for: Extracting chemical reactions from text using Snorkel
Source: BMC Bioinformatics. 2020 May 27;21:217. doi: 10.1186/s12859-020-03542-1 (PMC7251675; doi:10.1186/s12859-020-03542-1)
Supplement: Supplementary file 1 — Additional file 1. “Extracting Chemical Reactions from Text using Snorkel Supplemental Data”. File contains supplemental methods and results for the main manuscript. [file 12859_2020_3542_MOESM1_ESM.docx]

Extracting Chemical Reactions from Text using Snorkel

Supplemental Data

Emily K Mallory, Matthieu de Rochemonteix, Alex Ratner, Ambika Acharya, Chris Re, Roselie A Bright, Russ B Altman

EKM: Biomedical Informatics Training Program, Stanford University, Stanford, CA, USA

MdR: Department of Statistics, Stanford University, Stanford, CA, USA

RAB: Office of Health Informatics, Office of the Chief Scientist, Office of the Commissioner, Food and Drug Administration, Silver Spring, MD, USA

RBA: Departments of Medicine, Genetics, Bioengineering, and Biomedical Data Science, Stanford University, Stanford, CA, USA

AA, AR and CR: Department of Computer Science, Stanford University, Stanford, CA, USA

# Supplemental Data

This section gathers observations that are not directly relevant to this Snorkel application, but help the reader having a better understanding of the pipeline.

## Rebalancing procedure

The rebalancing procedure followed three steps:

- We chose a threshold on the marginals from the generative model (0.40) to divide all the training candidates into positive and negative candidates.
- We chose target class proportions (0.25).
- We kept the underrepresented class and sampled the overrepresented class to construct a dataset that matched the target proportions.

Given the class imbalance in the original dataset (5% of positive cases for the MetaCyc_Corpus, 1.5% for the Bacteria_Corpus), we were faced with situations where we sampled an incomplete subset of the variety found in the negative cases. While we expect that the labeling functions will not achieve perfect classification, the resulting errors in the training marginals may introduce bias in the rebalancing process.

For a given training set target size, the rebalancing parameter was roughly determined by the threshold: if we set a low threshold, we considered a lot of examples as positives, and sampled fewer examples from the negatives. However, the lower the threshold, the more false positives: the target class proportion was not actually met.

We used this analysis as a guideline to tune the generative model. The higher the precision of the generative model, the closer the proportion we got in the rebalanced dataset was close to the target proportion due to fewer false positives to sample for the target positive proportion. We therefore focused on a high precision to ensure full control of the rebalancing.

We included the class imbalance hyperparameters (target class proportion and class threshold) in the gridsearch to tune its parameters.

## Additional database exploration

The Bacteria_Corpus was very large, and contained a large variety of abstracts. We therefore performed an analysis of the dataset itself, before performing the extraction. The occurrences of some subsets of chemicals are particularly relevant to us in this work. This is a way to spot a potential structural difference between candidates that involve a chemical of interest and other candidates.

We were interested in two lists of drugs:

- Curated_set: A list of 50 drugs curated by Spanogiannopoulos et al. [1]
- FDA-approved: A list of 3325 FDA approved drugs curated by PharmGKB [2]

We defined subsets of the original dataset, by selecting sentences in which the drugs from a given list appear. Table 1 reports summary statistics for the number of candidates present in the three filter sets. We analyze the distribution of the number of candidates per sentence, that gives a good indication of the kind of sentence in which the chemicals from the list are found.

The average number of candidates per sentence is much higher for sentences that contain a chemical from either list. Actually, those terms mostly appear in enumerations of drugs, *i.e.,* sentences that have a lot of chemicals, hence a lot of candidates. We matched the candidates' chemicals and the chemicals from the reference list using MeSH [4], ChEBI identifiers [5] when available from the PubTator tags [6], and direct text matching otherwise.

Table 1. Summary statistics for the number of candidates with no entity filter, only FDA-approved chemicals, and curated drug set.

| Chemical filter | Sentences | Candidates | Mean | Median | 95% quantile |
| --- | --- | --- | --- | --- | --- |
| None | 1,106,386 | 8,936,941 | 8.077 | 2 | 30 |
| FDA-approved | 112,045 | 1,584,571 | 14.14 | 6 | 56 |
| Curated_set | 24,317 | 470,228 | 19.33 | 6 | 72 |

## Role of the development set

One of the specificities of the Snorkel pipeline is that the user does not need to curate examples, except for the development and test sets. The test set is out-of sample, so the only way the pipeline "sees" the gold labels is through the development set.

This subset of the data has a very particular role since we expected the labeling functions to be particularly adapted to the candidates of this subset. It is not simply a validation set that we use for the gridsearch. Consequently, analyzing the generative model performance on this dataset is particularly relevant, and is a good way to determine if the labeling functions have been properly designed.

We performed experiments with a different development set to confirm that it has a particular role. When the development set is not the one used to design the labeling functions, we found a slight decrease in the performance of all the models on the evaluation set.

## Advantage of the discriminative model over the generative model

Table 2 reports the performance of the generative model and the discriminative model when thresholded at $0.64$ on the two test sets. The $F_1$ score, precision and recall using a threshold do not reflect the overall complexity and performance of the models. We also report the Precision-Recall curves of the end models (discriminative and generative). We computed precision recall curves to assess the performances of the models (Figures 1 and 2). They show that the discriminative model has a **smoother precision-recall tradeoff**.

Table 2. Evaluation results on the Bacteria_Corpus - end models.

| Model | Evaluation set | Precision | Recall | F1 score |
| --- | --- | --- | --- | --- |
| Generative | Bacteria_Test | 0.92 | 0.28 | 0.43 |
|  | MetaCyc_Test | **1.00** | 0.33 | **0.49** |
| Discriminative  0.64 threshold | Bacteria_Test | 0.52 | **0.40** | 0.45 |
|  | MetaCyc_Test | 0.62 | 0.37 | 0.46 |


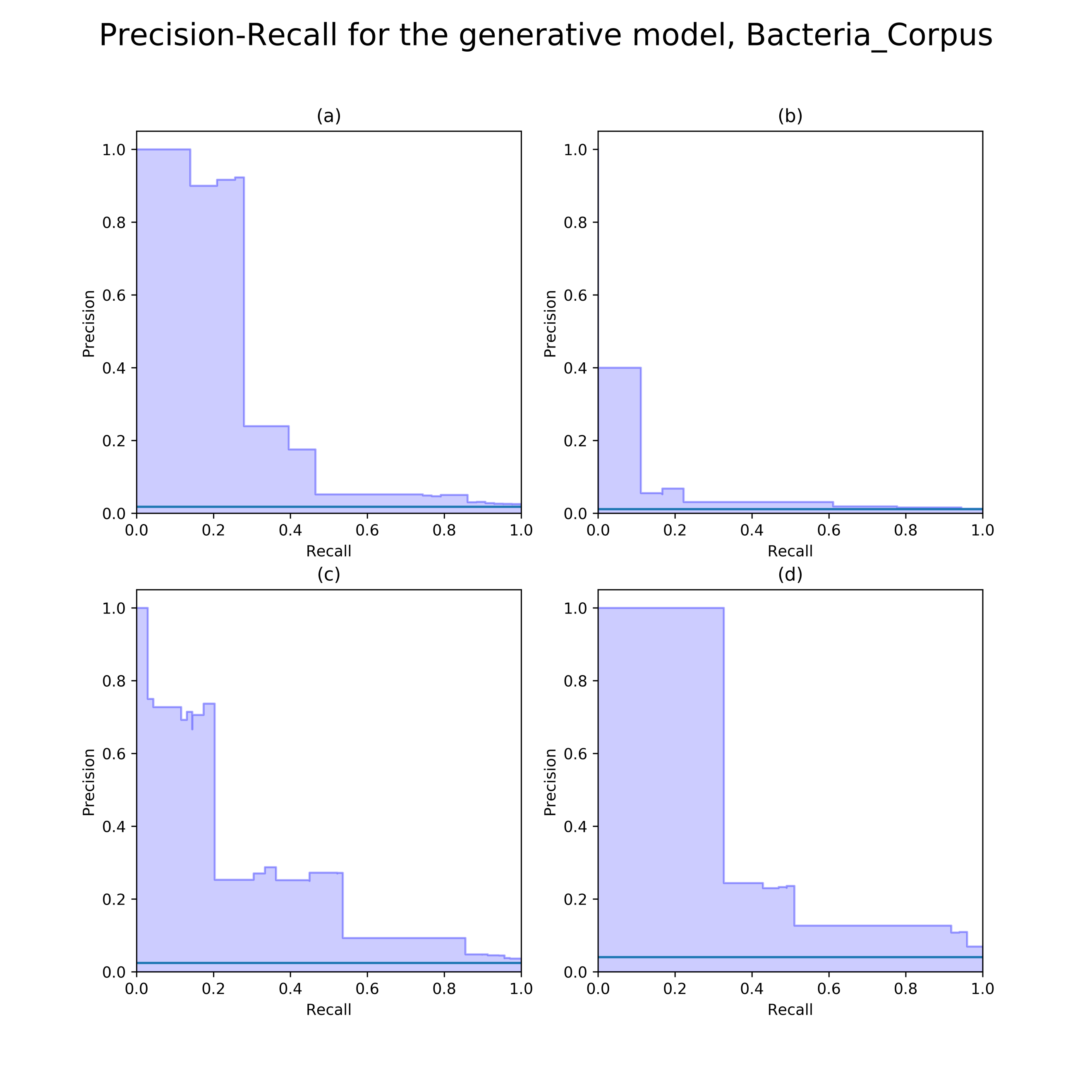


Figure 1. Precision-Recall (PR) curve and Average Precision (AP --- area under the PR curve) for the generative model on different evaluation sets. **(a)** PR for Bacteria_Test (AP = 0.33), **(b)** PR for Bacteria_Test2 (AP = 0.07), **(c)** PR for Bacteria_Dev (AP = 0.28), **(d)** PR for MetaCyc_Test (AP = 0.43). The blue line is the baseline performance, i.e., the performance of a classifier with random labels.


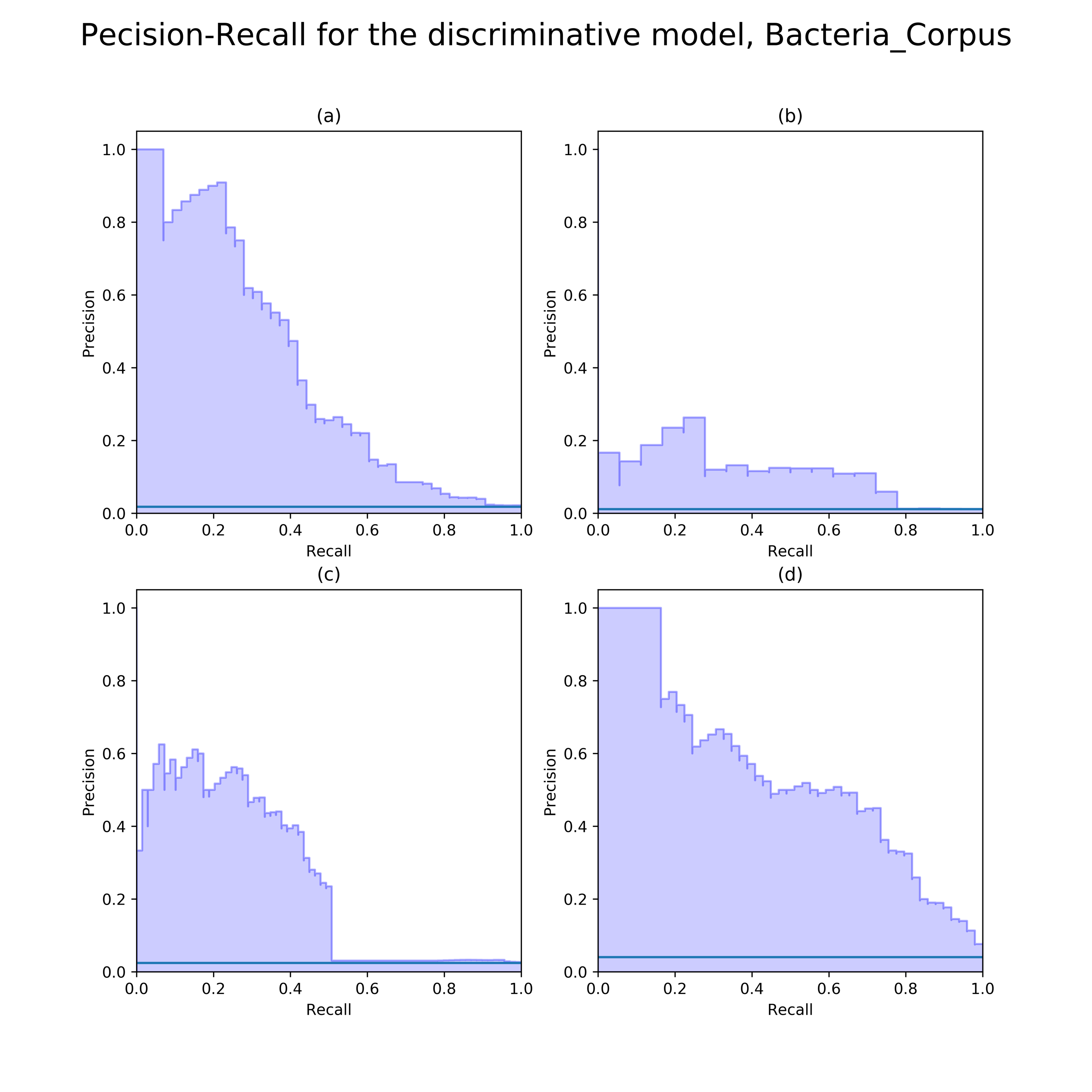


Figure 2. Precision-Recall (PR) curve and Average Precision (AP --- area under the PR curve) for the discriminative model on different evaluation sets. **(a)** PR for Bacteria_Test (AP = 0.40), **(b)** PR for Bacteria_Test2 (AP = 0.11), **(c)** PR for Bacteria_Dev (AP = 0.25), **(d)** PR for MetaCyc_Test (AP = 0.55). The blue line is the baseline performance, i.e., the performance of a classifier with random labels.

We explain this smoother behavior by the comparatively higher complexity of the features used by the discriminative model compared to the labeling functions. We observe that the precision-recall tradeoff of the generative model is **bimodal**: the precision is either very close to 1 or very close to 0. The discriminative model allows a much wider range of precision-recall tradeoffs. It is therefore much more adapted to subsequent applications, where we need the possibility to set the sensitivity of our classifier smoothly.

This also means that the score of the discriminative model can be used as a confidence score on the prediction. This is not the case with the generative model score, which is bimodal due to sparse reweighted labeling functions. The main advantage of the discriminative model in this case is not a lift in performance, but rather a much improved versatility in what the results may be used for, and much more robustness since it provides a meaningful confidence score on its predictions.

## Dataset rebalancing and class imbalance

### Motivation

Given the high computational cost of discriminative model training, we subsampled the training data used in the discriminative model. This procedure also handled the class imbalance, because we imposed the class proportions when rebalancing the dataset and trained the models with a larger proportion of positive examples than what is contained in the original dataset. We detail the complete rebalancing procedure in Supplemental Section 1.1

### Effect on the performance

Overall, the class imbalance was the main challenge and the biggest source of performance degradation between the MetaCyc_Corpus and the Bacteria_Corpus. The rebalancing process is a good way to tackle this, but it was deeply dependent on the performance of the generative model.

The experiments we performed showed that the optimal results were obtained by training on approximately 10% of the dataset. Actually, the negative examples were very similar to one another and did not bring much diversity in the training set. Training on more candidates was not only more expensive computationally, but also less performant.

Intuitively, leaving a lot of negative examples out of the training set and having selected positive examples from a generative model with high precision yields a dataset where the positive cases have high marginals, but some of them are absent. On the other hand, sampling more negative examples increases the number of positive examples in the training set that have low marginals.

When we have few positive examples that all have high marginals, we hope that the discriminative model uses them to capture meaningful features and will predict the missing positive examples correctly based on those features. However, if positives with low training marginals are included in the training set, the learning process is more difficult since there are errors in the labels.

In summary, we used the rebalancing as a way to tackle class imbalance, and also as a way to "clean" the training set and reduce the noise in the training marginals that are actually used by the discriminative model. However, we are ignoring a large part of the dataset in the discriminative model training due to this method. Using the training dataset more extensively could be a way to improve the overall performance.

## Model selection and perspectives

As discussed in Section 3.3.2, we used a different generative model for the Bacteria_Corpus. Tables 3 and 4 report the performance differences between the two models on respectively MetaCyc_Test and the relevant development set (MetaCyc_Dev and Bacteria_Dev), respectively.

Table 3. Comparison of generative model performance on the MetaCyc_Test subset.

| Corpus | Model | Precision | Recall | F1 score |
| --- | --- | --- | --- | --- |
| MetaCyc_Corpus | Gibbs | 0.79 | 0.22 | 0.34 |
|  | Matrix Completion | **0.83** | **0.29** | **0.43** |
| Bacteria_Corpus | Gibbs | 1.00 | 0.12 | 0.22 |
|  | Matrix Completion | **1.00** | **0.33** | **0.49** |

Table 4. Comparison of generative model performance on the development subsets (MetaCyc_Dev and Bacteria_Dev).

| Dev Subset | Model | Precision | Recall | F1 score |
| --- | --- | --- | --- | --- |
| MetaCyc_Dev | Gibbs | **0.84** | 0.35 | 0.49 |
|  | Matrix Completion | 0.82 | **0.38** | **0.52** |
| Bacteria_Dev | Gibbs | **0.83** | 0.07 | 0.13 |
|  | Matrix Completion | 0.44 | **0.36** | **0.40** |

Matrix completion brings a significant performance lift on both corpora, for the test set. However, the performance lift on the development set for the MetaCyc corpus is only $.03$ in recall, with a precision decrease. Given that we are only allowed to use the development set to choose the model and given that the matrix completion method was still in the development phase during development and evaluation of the chemical reaction application, we kept the Gibbs Sampling method for the MetaCyc_Corpus.

For the discriminative model, the Snorkel pipeline offers two different end models:

- A recurrent neural network model -- Long Short Term Memory (LSTM).
- A logistic regression -- that uses a set of *traditional* NLP features (n-grams, etc...), and a logistic regression as the end classifier.

We trained and evaluated the LSTM model, that performed slightly worse and was less robust than logistic regression. We kept the logistic regression model for the final evaluation.

## Labeling Functions

Table 5. Labeling functions for the MetaCyc_Corpus

| Labeling function | Description |
| --- | --- |
| LF_to_from_and | If the chemicals are separated by only one word, and if this word is “to” or “from”, returns TRUE or FALSE depending on the orientation of the candidate. Also handles the case where there are 3 words in between, with a chemical in the middle, and the other words are “and” and (“to” or “from”) in a similar way |
| LF_keyword_context | If there is a word of a given list, such as *reduce, oxidize, transform o*r *afford* between the two words, we label TRUE |
| LF_sep_verb | If the chemicals are separated by a verb, we label TRUE |
| LF_sep_adverb | If the chemicals are separated by an adverb, we label FALSE |
| LF_redox | If the chemicals are separated by a sequence of words that contains *oxidized to*, *reduced to*, *conversion to*, we label TRUE |
| LF_argument_order | If the candidate product is before the candidate substrate, we label FALSE |
| LF_sep_and | If the chemicals are separated by *and*, we label FALSE |
| LF_followed_by_noun | If one of the chemicals is followed by a noun, we label FALSE |
| LF_followed_ase | If one of the chemicals is followed by a word that ends with *-ase*, we label FALSE |
| LF_general_chemical | If one of the chemicals is in a list of common chemical terms such as *amino acid, sugar, adenosine, amide, adenine*, *etc*..., we label FALSE |
| LF_adjacent_mentions | If the chemicals are adjacent, we label FALSE |
| LF_gene_words | If one of the chemicals is in a list of genetic terms such as *reductase, dna, gene*, we label FALSE |
| LF_sep_or | If the chemicals are separated by *or*, we label FALSE |
| LF_sep_comma | If the chemicals are separated by a comma, we label FALSE |
| LF_sep_via | If the chemicals are separated by *via, in, etc*, we label FALSE |
| LF_sep_sym | If the chemicals are separated by a single character, we label FALSE |

Table 6. Additional labeling functions on the Bacteria_Corpus

| Labeling function | Description |
| --- | --- |
| LF_metacyc | If the chemical reaction is already in the MetaCyc curated database, we label TRUE |
| LF_chemical_element | If one of the chemicals is a chemical element, we label FALSE (as it is likely to be a catalyst) |
| LF_chemical_symbol | If one of the chemicals is a chemical symbol, we label FALSE |
| LF_group | If there is a close mention of a functional chemical group, we label FALSE (as it is more likely descriptive of a structure than of a reaction) |
| LF_treatment | If there is mention of keywords frequently associated with clinical trials, we label FALSE |
| LF_external_action | If there is mention of the effect on/of an external, organism, through words like *inhibit, resistant, utilizing, transport, stimulate, etc...* we label FALSE |

Table 7. Example sentences for MetaCyc_Corpus labeling functions. Potential substrates are denoted by red and potential example products are denoted by blue. Non-specific chemical entities are denoted by single letters.

| Labeling function | Example | Outcome |
| --- | --- | --- |
| LF_to_from_and | The reaction produced A from C and D. | TRUE |
| LF_to_from_and | The reaction produced A from D. | FALSE |
| LF_to_from_and | The reaction produced A from the compounds C, B, and D. | ABSTAIN |
| LF_keyword_context | A was reduced to B. | TRUE |
| LF_keyword_context | A was reduced to B. | ABSTAIN |
| LF_sep_verb | A affords B. | TRUE |
| LF_sep_verb | A affords B. | ABSTAIN |
| LF_sep_adverb | The degradation of A by B. | FALSE |
| LF_sep_adverb | The degradation of A by B. | ABSTAIN |
| LF_redox | A is reduced to B. | TRUE |
| LF_argument_order | A is produced by B. | TRUE |
| LF_argument_order | A is produced by B. | FALSE |
| LF_sep_and | C produces A and B. | FALSE |
| LF_sep_and | C produces A and degrades B. | ABSTAIN |
| LF_followed_by_noun | B is involved in the A pathway. | FALSE |
| LF_followed_ase | A is degraded by the B reductase. | FALSE |
| LF_general_chemical | … has an effect on the degradation of uridine through A. | FALSE |
| LF_adjacent_mentions | The adjunction of copper sulfate to the solution. | FALSE |
| LF_gene_words | A has an effect on several human proteins. | FALSE |
| LF_sep_or | We studied the effects of the adjunction of A or B to the cultures on… | FALSE |
| LF_sep_comma | The process afforded A, B, C, D, and E. | FALSE |
| LF_sep_via | The transformation of A to B via C. | FALSE |
| LF_sep_sym | In this study, we model the kinetics of the AxB reaction. | FALSE |

Table 8. Example sentences for Bacteria_Corpus labeling functions. Potential substrates are denoted by red and potential example products are denoted by blue. Non-specific chemical entities are denoted by single letters.

| Labeling function | Example | Outcome |
| --- | --- | --- |
| LF_metacyc | The degradation of C-3-NONENAL into 4-HYDROPEROXYOCTADECAT-2-NONENAL… | TRUE |
| LF_chemical_element | We measured nitrogen and B concentrations. | FALSE |
| LF_chemical_symbol | In those conditions, Pt is a known catalyst of the degradation of B. | FALSE |
| LF_group | B was produced after the removal of A from C. | FALSE |
| LF_treatment | In this study, we examine the production of A in patients exposed to B. | FALSE |
| LF_external_action | It is well known that exposure to A stimulates the production of B. | FALSE |

# References

1. Spanogiannopoulos, P., et al., *The microbial pharmacists within us: a metagenomic view of xenobiotic metabolism.* Nat Rev Microbiol, 2016. **14**(5): p. 273-87.

2. Whirl-Carrillo, M., et al., *Pharmacogenomics knowledge for personalized medicine.* Clin Pharmacol Ther, 2012. **92**(4): p. 414-7.

3. Krallinger, M., et al. *Overview of the BioCreative VI chemical-protein interaction track*. in *Proceedings of the BioCreative VI challenge evaluation workshop*. 2017.

4. Lipscomb, C.E., *Medical Subject Headings (MeSH).* Bull Med Libr Assoc, 2000. **88**(3): p. 265-6.

5. Hastings, J., et al., *The ChEBI reference database and ontology for biologically relevant chemistry: enhancements for 2013.* Nucleic Acids Res, 2013. **41**(Database issue): p. D456-63.

6. Wei, C.H., H.Y. Kao, and Z. Lu, *PubTator: a web-based text mining tool for assisting biocuration.* Nucleic Acids Res, 2013. **41**(Web Server issue): p. W518-22.

7. Peasland, A., et al., *Identification and evaluation of a potent novel ATR inhibitor, NU6027, in breast and ovarian cancer cell lines.* Br J Cancer, 2011. **105**(3): p. 372-81.
